# Supplementary material for: Psychosocial correlates of safe sex self-efficacy among in-school adolescent girls in Lagos, Nigeria
Source: PLoS One. 2020 Jun 23;15(6):e0234788. doi: 10.1371/journal.pone.0234788 (PMC7310695; doi:10.1371/journal.pone.0234788)
Supplement: S1 File — (PDF) [file pone.0234788.s001.pdf]

JEWEL Intervention to improve health outcomes among adolescent girls and young women in Nigeria.

Introduction: You have been invited to participate in a program that is intended to reduce the incidence of HIV among young women in Nigeria and improve overall health and well-being as well as livelihood opportunities through jewelry making. It is called JEWEL (Jewelry Enterprises for young Women Empowering their Lives). Your answers to the question below are to help us improve the program. Your answers to the questions we ask will be kept confidential, in other words, no one will be able to trace it back to you. Thank you for your participation.

Section 0: Background

No.	Questions and Filters	Coding Categories
Q001	Are you able to provide informed consent (i.e. you do not have a permanent physical disability (e.g. deafness) or mental disability which prevents you from providing consent to participate in this study)?	PLEASE CIRCLE YOUR ANSWER BELOW<br>1) Yes 2) NO
Q002	*My sex or gender is*	PLEASE CIRCLE ANSWER BELOW<br>1) Male 2) Female
Q003	How old are you?	__ __ YEARS
Q004	What level are you in school?	
Q005	What is your ethnic background?	
Q006	What is your religion?	
Q007	Is there an adult 25 years or older who you consider to be your mentor? That is, someone you can go to for support and guidance or if you need to make an important decision, or who inspires you to do your best?"	1) YES 2) NO
Q008	If you answered yes to Q007, "What is his/her relationship to you?"	THE PERSON IS MY______________________
Q009	What is the name of your school?	
Q010	Which local government area do you live in?	

Section 1: Future Orientation

No.	Questions and Filters	Coding Categories (Please circle only one of the response)			
Q101	What happens to me in the future mostly depends on me	1) Never True	2) Sometime True	3) Often True	4) Always True
Q102	I can see my life 10 years from now.	1) Never True	2) Sometime True	3) Often True	4) Always True
Q103	I think often about what tomorrow may bring.	1) Never True	2) Sometime True	3) Often True	4) Always True
Q104	Before making a decision I weigh the good versus the bad.	1) Never True	2) Sometime True	3) Often True	4) Always True
Q105	I just live for today	1) Never True	2) Sometime True	3) Often True	4) Always True
Q106	I can do just about anything I really set my mind to do	1) Never True	2) Sometime True	3) Often True	4) Always True
Q107	It's really no use worrying about the future, because what will be will be	1) Never True	2) Sometime True	3) Often True	4) Always True
Q108	My future is what I make of it	1) Never True	2) Sometime True	3) Often True	4) Always True
Q109	I have great faith in the future	1) Never True	2) Sometime True	3) Often True	4) Always True
Q110	Sometimes I feel there is nothing to look forward to in the future	1) Never True	2) Sometime True	3) Often True	4) Always True

Section 2: HIV/AIDS Knowledge

No.	Questions and Filters	Coding Categories (Please circle only one response)
Q201	Most people who have the AIDS virus look sick.	1) True 2) False 3) Don't Know
Q202	You can get the AIDS virus during oral sex.	1) True 2) False 3) Don't Know
Q203	A person can get the AIDS virus in one sexual contact.	1) True 2) False 3) Don't Know
Q204	Keeping in good physical shape is the best way to keep from getting AIDS.	1) True 2) False 3) Don't Know
Q205	Condoms make intercourse completely safe.	1) True 2) False 3) Don't Know
Q206	A shower after sex reduces the risk of getting AIDS.	1) True 2) False 3) Don't Know
Q207	When people don't have other partners, they don't need to practice safe sex.	1) True 2) False 3) Don't Know
Q208	Oral sex is safe if partners don't swallow.	1) True 2) False 3) Don't Know
Q209	People who have the AIDS virus quickly get sick.	1) True 2) False 3) Don't Know
Q210	By having just one sex partner at a time you can protect yourself from AIDS.	1) True 2) False 3) Don't Know
Q211	The AIDS virus doesn't go through unbroken skin.	1) True 2) False 3) Don't Know
Q212	Anal (rectal) intercourse is risky because it transmits the AIDS virus.	1) True 2) False 3) Don't Know
Q213	A person must have a lot of different sex partners to be at risk for AIDS.	1) True 2) False 3) Don't Know
Q214	The AIDS virus can be transmitted by mosquitoes or cockroaches	1) True 2) False 3) Don't Know
Q215	If the man pulls out (withdraws) before orgasm, then intercourse is safe.	1) True 2) False 3) Don't Know
Q216	A good diet and plenty of sleep will keep a person from getting AIDS.	1) True 2) False 3) Don't Know
Q217	A negative result on the HIV test can happen even if somebody has the AIDS virus.	1) True 2) False 3) Don't Know
Q218	It's more important for people to protect themselves against AIDS in big cities than in small cities.	1) True 2) False 3) Don't Know
Q219	Only receptive anal sex transmits AIDS.	1) True 2) False 3) Don't Know
Q220	Most people who have the AIDS virus know they have it.	1) True 2) False 3) Don't Know
Q221	No case of AIDS was ever caused by social (dry) kissing.	1) True 2) False 3) Don't Know
Q222	All sexually transmitted diseases can be cured.	1) True 2) False 3) Don't Know
Q223	Cum (semen) carries the AIDS virus.	1) True 2) False 3) Don't Know
Q224	Sharing toothbrushes and razors can transmit the AIDS virus.	1) True 2) False 3) Don't Know
Q225	Have you ever tested for HIV?	1) Yes 2) No

Section 3: Self-Efficacy

No.	Questions and Filters	Coding Categories (Please circle only one response)
Q301	How sure are you that would be able to say NO to having sexual intercourse: With someone you have known for few days or less	1) Not at all sure 2) A little sure 3) Sure 4) Very sure

Q302	How sure are you that would be able to say NO to having sexual intercourse: With some whose sexual relationship history is not known to you	1) Not at all sure	2) A little sure	3) Sure	4) Very sure
Q303	How sure are you that would be able to say NO to having sexual intercourse: With someone you have dated for a long time?	1) Not at all sure	2) A little sure	3) Sure	4) Very sure
Q304	How sure are you that would be able to say NO to having sexual intercourse: With someone you want to date?	1) Not at all sure	2) A little sure	3) Sure	4) Very sure
Q305	How sure are you that would be able to say NO to having sexual intercourse: With someone who you want to fall in love with you?	1) Not at all sure	2) A little sure	3) Sure	4) Very sure
Q306	How sure are you that would be able to say NO to having sexual intercourse: With someone who is pushing you to have sexual intercourse?	1) Not at all sure	2) A little sure	3) Sure	4) Very sure
Q307	I feel sure that: I could talk about using a condom with any potential partner	1) Not at all sure	2) A little sure	3) Sure	4) Very sure
Q308	I feel sure that: I could talk about using condoms if I was unsure about my partner's feelings about condoms.	1) Not at all sure	2) A little sure	3) Sure	4) Very sure
Q309	I feel sure that: I could have an intimate relationship with only one person for a long time	1) Not at all sure	2) A little sure	3) Sure	4) Very sure
Q310	I feel sure that: I could say no to sex if my partner refused to use a condom	1) Not at all sure	2) A little sure	3) Sure	4) Very sure
Q311	I feel sure that: I could talk about using condoms with a potential sexual partner before we started to hug and kiss	1) Not at all sure	2) A little sure	3) Sure	4) Very sure
Q312	I feel sure that: I could say no to sex	1) Not at all sure	2) A little sure	3) Sure	4) Very sure
Q313	I feel sure that: I could discuss preventing AIDS with a potential partner	1) Not at all sure	2) A little sure	3) Sure	4) Very sure
Q314	I feel sure that: I could use a condom correctly	1) Not at all sure	2) A little sure	3) Sure	4) Very sure
Q315	I feel sure that: Use a condom every time I have sexual intercourse	1) Not at all sure	2) A little sure	3) Sure	4) Very sure

Section 4: Sense of Community and Social Support

No.	Questions and Filters	Coding Categories (Please circle only one response)

Q401	I feel sense of contact with people who care for me	1) Strongly Agree 2) Agree 3) Disagree 4) Strongly Disagree
Q402	I feel connected with others who are important to me	1) Strongly Agree 2) Agree 3) Disagree 4) Strongly Disagree
Q403	I feel that I can talk about my problems with my family	1) Strongly Agree 2) Agree 3) Disagree 4) Strongly Disagree
Q404	I feel that there is at least someone with whom I can share my most private thoughts.	1) Strongly Agree 2) Agree 3) Disagree 4) Strongly Disagree
Q405	There is at least one person I know whose advice I really trust	1) Strongly Agree 2) Agree 3) Disagree 4) Strongly Disagree
Q406	I feel that I can talk about my problems with my friends	1) Strongly Agree 2) Agree 3) Disagree 4) Strongly Disagree
Q407	I feel that I can ask adults for advice about HIV or other sexually transmitted diseases	1) Strongly Agree 2) Agree 3) Disagree 4) Strongly Disagree
Q408	I feel that I have adults who listen to me	1) Strongly Agree 2) Agree 3) Disagree 4) Strongly Disagree

Section 5: Monitoring of leisure time after school

No.	Questions and Filters	Coding Categories (Please circle only one response)
Q501	How much does your mother/father/guardian really know about: Who your friends are?	1) Knows a lot 2) Knows a little 3) Doesn't know anything
Q502	How much does your mother/father/guardian really know about: How you spend your money?	1) Knows a lot 2) Knows a little 3) Doesn't know anything
Q503	How much does your mother/father/guardian really know about: Where you are after school?	1) Knows a lot 2) Knows a little 3) Doesn't know anything
Q504	How much does your mother/father/guardian really know about: Where you go at night?	1) Knows a lot 2) Knows a little 3) Doesn't know anything
Q505	How much does your mother/father/guardian really know about: What you do with your free time?	1) Knows a lot 2) Knows a little 3) Doesn't know anything
