# Supplementary material for: Psychosocial correlates of safe sex self-efficacy among in-school adolescent girls in Lagos, Nigeria
Source: PLoS One. 2020 Jun 23;15(6):e0234788. doi: 10.1371/journal.pone.0234788 (PMC7310695; doi:10.1371/journal.pone.0234788)
Supplement: Attachment — Submitted filename: Plos One Review 12.3.docx [file pone.0234788.s002.docx]

**“Psychosocial correlates of safe sex self-efficacy among in-school adolescent girls in**

**Lagos, Nigeria**" is a paper whose topic is likely to be of interest to the readership of PLoS One. The current study examined how social support, parental monitoring, and future orientation influence perceived safe sex self-efficacy among in-school adolescent girls in Nigeria. This study contributes to the literature by examining correlates of safe sex self-efficacy among a unique population. However, I have several concerns about the lack of information regarding the rationale for the study, the omission of sexual behavior data from the models, and the inferences made in the discussion. If these points of clarification can be addressed, I would recommend resubmitting to PLos One for publication.

Central Concerns:

1. My overarching concern for this paper involves its lack of rationale. While it is clear that adolescent girls and young women (AGYW) have high rates of HIV an STIs, it is not clear how this paper moves the research forward or what theoretical or conceptual base was used to develop the research question.
2. There has been much research done on correlates of sexual self-efficacy. How is the paper different? How are Nigerian AGYW different from other populations and why is this research needed?
3. What is the theoretical rationale for including all these measures in the models? It seems like the Theory of Planned Behavior or the Theory of Planned Behavior and Reasoned Action may be guiding this work, but the inclusion of social support and parental monitoring makes this unclear. Please clarify.
4. The fact that there is no data on whether or not the AGYW in the study were sexually active is a major limitation. Those who are sexually active are likely different than those who are not - especially in terms of safe sex self-efficacy. If this data was collected it should be entered as a covariate. If not, these findings are quite limited.

Other points

5. In the introduction, the second paragraph is disjointed. The point of the paragraph is to discuss predictors of self-efficacy. Therefore, the section should first discuss why self-efficacy is important in predicting sexual behavior. Then discuss the associations between the other variables and self-efficacy. This section should end with a stronger rationale for examining these associations among AGYW in Nigeria.
6. Safe sex negotiation is different from self-efficacy. Therefore, the authors should either remove the reference to safe sex negotiation or integrate it into the conceptual framework and analysis.
7. The authors should elaborate on why each independent variable may be related to safe sex self-efficacy – placing emphasis on if these associations have been demonstrated among AGYW and/or AGYW in Nigeria. The introduction should also explain why it is important to look at these associations among AGYW in Nigeria.
8. Please elaborate on why it is important for research to be conducted at schools (e.g., school-based interventions).
9. Please describe your randomization method.
10. Please describe how parental consent was obtained.
11. Please describe how survey was administered (e.g., read aloud).
12. Please give the American, English, and other relevant equivalents to Senior Secondary School.
13. Please include a meaningful cut-off or norm score for the measures in Table 2. For example, what score represents average knowledge of HIV?
14. Please clarify whether age was modeled as a continuous or categorial variable. It should be modeled as a continuous variable.
15. Typically, variables that are not related to the outcome in the bivariate analysis are not included in the multivariate model. Therefore, please provide a rationale for including all the demographic and psychosocial factors in the models.
16. The association between religion and self-efficacy is an interesting finding. Perhaps you should conduct the bivariate analysis separated by type of school? No table would be necessary. It would just be to identify important correlates of each model.
17. In the discussion the authors state:

“Thus, while HIV knowledge may not be associated with risky sexual behaviors, it could potentially be indirectly associated with risky sexual behaviors and mediated by safe sex self-efficacy. While this study did not examine actual sexual behavior, this potential mediating relationship should be considered in future studies.

This mediating relationship is the impetus for several theories. Perhaps the authors should describe how this relationship may be different for this population.

18. The findings related to future orientation are quite important! It is also important for interventions to understand future orientation for HIV prevention interventions.
19. “Protective” language is too strong without having conducted a moderation analyses - see Rutter's work on resilience

In sum, I think the authors need to revisit explain the rationale for their study, strengthen the methodology, and refine the discussion. Thank you for the opportunity to provide feedback on this very interesting manuscript.

Decision: Revised and Resubmit
