# Supplementary material for: Psychosocial correlates of safe sex self-efficacy among in-school adolescent girls in Lagos, Nigeria
Source: PLoS One. 2020 Jun 23;15(6):e0234788. doi: 10.1371/journal.pone.0234788 (PMC7310695; doi:10.1371/journal.pone.0234788)
Supplement: Attachment — Submitted filename: Plos One Review 12.3_Updated.docx [file pone.0234788.s004.docx]

**School for Public Health and Social Justice**

Salus Center

3545 Lafayette Avenue

Saint Louis, MO 63103

Email: [nwaozur2@gmail.com](mailto:nwaozur2@gmail.com)

Dear Dr. Wendee Wechsberg,

We appreciate the time and effort that you and the reviewers have dedicated to providing additional feedback to the manuscript: **Psychosocial correlates of safe sex self-efficacy among in-school adolescent girls in Lagos, Nigeria** (**PONE-D-19-29960R1**). We have been able to incorporate changes to reflect most of the suggestions provided by the reviewers. Here is a point-by-point response to the reviewers’ comments and concerns (please note that responses to the comments are in BOLD).

**Comments to the Author**

1. If the authors have adequately addressed your comments raised in a previous round of review and you feel that this manuscript is now acceptable for publication, you may indicate that here to bypass the “Comments to the Author” section, enter your conflict of interest statement in the “Confidential to Editor” section, and submit your "Accept" recommendation.

Reviewer #1: (No Response)
Reviewer #2: All comments have been addressed

**Response: Thank you for the kind response.**

2. Is the manuscript technically sound, and do the data support the conclusions?

The manuscript must describe a technically sound piece of scientific research with data that supports the conclusions. Experiments must have been conducted rigorously, with appropriate controls, replication, and sample sizes. The conclusions must be drawn appropriately based on the data presented.

Reviewer #1: Yes
Reviewer #2: Yes

**Response: Thank you for the kind response.**

3. Has the statistical analysis been performed appropriately and rigorously?

Reviewer #1: Yes
Reviewer #2: Yes

**Response: Thank you for the kind response.**

4. Have the authors made all data underlying the findings in their manuscript fully available?

The [PLOS Data policy](http://www.plosone.org/static/policies.action#sharing) requires authors to make all data underlying the findings described in their manuscript fully available without restriction, with rare exception (please refer to the Data Availability Statement in the manuscript PDF file). The data should be provided as part of the manuscript or its supporting information, or deposited to a public repository. For example, in addition to summary statistics, the data points behind means, medians and variance measures should be available. If there are restrictions on publicly sharing data—e.g. participant privacy or use of data from a third party—those must be specified.

Reviewer #1: Yes
Reviewer #2: Yes

**Response: Thank you for the kind response.**

 5. Is the manuscript presented in an intelligible fashion and written in standard English?

PLOS ONE does not copyedit accepted manuscripts, so the language in submitted articles must be clear, correct, and unambiguous. Any typographical or grammatical errors should be corrected at revision, so please note any specific errors here.

Reviewer #1: Yes
Reviewer #2: Yes

**Response: Thank you for the kind response.**  
6. Review Comments to the Author

Please use the space provided to explain your answers to the questions above. You may also include additional comments for the author, including concerns about dual publication, research ethics, or publication ethics. (Please upload your review as an attachment if it exceeds 20,000 characters)

Reviewer #1: (No Response)

Reviewer #2: While, the authors have addressed the comments of the reviewers. The manuscript needs to be thoroughly edited. Furthermore, the introduction of the manuscript still needs substantial improvement.

The first paragraph is entirely too long and disjointed. The authors should redevelop the introduction so that it comprises separate and coherent paragraphs with the following:

Paragraph 1 -introduction to the problem
Paragraph 2 - Previous research examining the problem
Paragraph 3 - Theoretical Framework for understanding the problem
Paragraph 4 - Current study

This explanation of the RAA is a great start. However, the authors should elaborate on the major points of the RAA and then situate the constructs of the current study in this framework.

**We appreciate the helpful feedback. We have revised the manuscript introduction to reflect the reviewer’s feedback. We restructured the introduction to be concise and include the following information: introduction to the problem, previous research examining the problem, theoretical framework for understanding the problem and the current study.**

7. PLOS authors have the option to publish the peer review history of their article ([what does this mean?](https://journals.plos.org/plosone/s/editorial-and-peer-review-process#loc-peer-review-history)). If published, this will include your full peer review and any attached files.


If you choose “no”, your identity will remain anonymous but your review may still be made public.

**Do you want your identity to be public for this peer review?** For information about this choice, including consent withdrawal, please see our [Privacy Policy](https://www.plos.org/privacy-policy).

Reviewer #1: No
Reviewer #2: No

Sincerely,

Ucheoma Nwaozuru, MSc
